# Food Biodiversity and its Association with Diet Quality and Health Outcomes-A Scoping Review

**DOI:** 10.1016/j.advnut.2025.100551

**Published:** 2025-11-01

**Authors:** Jinke H Baan Hofman, Celia Bannenberg Cavero, Mariska Dötsch-Klerk, Anne J Wanders, Corné van Dooren, Edith JM Feskens, Baukje de Roos, Sander Biesbroek

**Affiliations:** 1 Nutrition and Health, Unilever Foods Innovation Centre, Wageningen, The Netherlands; 2 Division of Human Nutrition and Health, Wageningen University & Research, Wageningen, The Netherlands; 3 World Wide Fund for Nature Netherlands, Zeist, The Netherlands; 4 The Rowett Institute, University of Aberdeen, Aberdeen, United Kingdom

**Keywords:** food biodiversity, nutrition, human, diet quality, health outcomes, sustainability, metrics, dietary species richness

## Abstract

Food biodiversity is receiving increased attention because of global food systems being a major contributor to biodiversity loss. Food biodiversity, defined as the diversity of plants, animals, and other organisms used for food, may benefit both human and planetary health. Despite this potential, little is known about the association between food biodiversity and the healthiness of human diets. This systematic scoping review presents an overview of the nexus between food biodiversity, diet quality, health outcomes, and environmental impact. Three search strategies were performed in Scopus and PubMed Central, to identify English articles published up until December 2024 on food biodiversity in relation to diet quality, health outcomes, and environmental impact. Eight studies reported on the association between food biodiversity and diet quality, and 4 on the association between food biodiversity and health outcomes. No studies reported on the association between food biodiversity and environmental impact. The studies quantified food biodiversity using Nutritional Functional Diversity, Dietary Species Richness (DSR), Simpson Diversity Index, Shannon Diversity Index, Berger-Parker Index, or a combination of these. One study compared the latter 4 metrics by calculating Hill numbers. Despite using different metrics, all studies showed significant positive associations between food biodiversity and nutritional adequacy, a reduced risk of total and cause-specific mortality, or a reduced risk of gastrointestinal cancers. One study reported a nonsignificant association between DSR and body fat percentage. In conclusion, limited available studies consistently find a positive association between food biodiversity, diet quality, and decreased health risks, highlighting the potential of food biodiversity to improve the healthiness of diets. Currently, DSR is proposed to be the most feasible metric to quantify food biodiversity. Future studies should focus on the added value of food biodiversity over dietary diversity in relation to human and planetary health, which is currently unclear.

**Statements of Significance**

This scoping review consolidates published insights into the association between the concept of food biodiversity and diet quality and health outcomes. It identifies knowledge gaps, offering an enhanced understanding of areas that require further research. In addition, this review evaluates food biodiversity quantification methodologies to promote consistent use and enable comparability across future studies.

## Introduction

Within the context of global food systems, agriculture, and human nutrition, the world is facing 2 key challenges. The first is to ensure that everyone has access to a healthy diet, thereby reducing the double burden of malnutrition present in many parts of the world, characterized by the coexistence of undernutrition along with overweight, obesity, or diet-related noncommunicable diseases [1]. The second is how to feed a growing global population in a way that is sustainable for our planet. The current global food production system accounts for ∼25% of greenhouse gas emissions, uses 40% of all habitable land, and accounts for 70% of freshwater use [2]. It thus contributes to climate change and environmental degradation, including biodiversity loss [3]. Therefore, food systems require substantial transformation to ensure both human and planetary health [4].

Food system transformation involves not only adapting and improving food production methods to produce healthier products with minimal environmental impact, but also changing consumption habits toward healthier and sustainable diets. Our diets can be a key lever in addressing both human and planetary health issues. This includes shifting toward more plant-based and diverse diets that have less detrimental impacts on our planet. It is a 2-way relationship: human diets impact the ecosystem’s health, including biodiversity loss, whereas simultaneously the ecosystems’ health impacts the healthfulness and nutritional quality of our diets. This is encompassed in the definition of sustainable diets, which states: “Sustainable diets are those diets with low environmental impacts which contribute to food and nutrition security, and to healthy life for present and future generations. Sustainable diets are protective and respectful of biodiversity and ecosystems, (…) nutritionally adequate, safe, and healthy; (…)” [5].

At the same time, 1 of the 4 principles of a healthy diet is diversity, alongside adequacy, balance, and moderation [6]. A diverse diet composed of a large variety of foods, consumed both between and within food groups, contributes to meeting micronutrient requirements, and is protective of noncommunicable diseases [6,7]. Measures of dietary diversity, which estimate food consumption on a food group level, have often been used as an indicator of diet quality, especially in low- and middle-income countries (LMICs) [8]. Moreover, most food-based dietary guidelines promote eating a varied diet. However, despite the large number of food species available worldwide, only 9 food species account for over 66% of the global crop production for human consumption by weight [9]. The decline in biodiversity in nature and agricultural ecosystems results in a decline of animal and plant species and a decrease in the genetic diversity of diets, respectively. The diversity of plants, animals, and other organisms used for human food consumption, both cultivated and from the wild, is defined as food biodiversity, a concept receiving growing attention as it encompasses the notions of biodiversity in nature, agriculture, and human diets [7].

The concept of food biodiversity reflects the diversity in a diet on a more granular level, referring to the diversity in food species consumed, rather than diversity between or within food groups. The concept of food biodiversity emerged from the discipline of ecology, where species diversity in natural or agricultural ecosystems is measured in terms of species richness and abundance, species evenness (relative distribution), and species functional (dis)similarity. When applied within nutrition sciences, food biodiversity covers similar dimensions in measuring diversity.

Food biodiversity has been identified as a potential lever to improve human and planetary health simultaneously, but there are still multiple unknowns that need to be addressed to understand the role of this concept in optimizing both [10]. Therefore, this scoping review aims to provide a systematic overview of available data on the association between food biodiversity and diet quality, health outcomes, and environmental impact, as well as to evaluate the different methods currently used to quantify food biodiversity.

## Methods

The approach for this scoping review was based on the Preferred Reporting Items for Systematic Reviews and Meta-Analyses Extension for Scoping Reviews (PRISMA-ScR) guidelines [11]. Three search strategies were developed to find articles on food biodiversity related to diet quality (search 1), health outcomes (search 2), and environmental impact (search 3), respectively. The search strategies were refined through team discussions. The final search strategies are summarized in Supplemental Table 1. The searches were performed in Scopus and either PubMed Central (searches 1 and 2) or PubMed (search 3). Additionally, reference lists of included studies were screened to identify additional relevant studies.

### Eligibility criteria

Inclusion and exclusion criteria for title, abstract, and full-text screening were based on the Population, Intervention, Comparators, Outcomes, and Study criteria (Table 1). In brief, a study was eligible when it used a quantitative measure for food biodiversity, diet quality, selected health outcomes, or environmental impact indicators. Focusing on quantitative outcomes allowed for measurability and comparison. The search was limited to articles published in English in peer-reviewed journals before the end of December 2024. The reviewers were aware of 2 articles being published after this date, and these are included as “other sources.”

**TABLE 1:** Inclusion and exclusion criteria for title, abstract, and full-text screening were based on the PICOS model.

Parameter	Description
Population	Inclusion criteria: studies on human individuals of any age, without geographical limitations
Interventions	Inclusion criteria: any study measuring participants’ intake of diversity of the diet on a species level
Comparators	Any or none
Outcomes	Inclusion criteria for search 1: any measure of diet quality or nutrient adequacy, measured on an individual level Inclusion criteria for search 2: selected measures of physical and mental health outcome, intermediate biomarker of health or mortality, measured on an individual level Inclusion criteria for search 3: environmental impact indicators of biodiversity loss, greenhouse gas emissions, land use, freshwater use, eutrophication, and/or acidification
Study design	Inclusion criteria: all original studies with any study design were considered [eg, randomized control trials, cross-sectional, (prospective) cohort, and case-control]. Reviews were used to identify potentially relevant studies from its reference list Exclusion criteria: reviews, editorials, conference reports, comments, letters, protocols, and expert opinions

Abbreviation: PICOS, Population, Intervention, Comparators, Outcomes, and Study.

### Selection process

Each systematic search was developed and conducted by a primary reviewer (JHBH or CBC). A second reviewer (JHBH, CBC, or MD-K) repeated the searches and screened ≥10% of search results. After removing duplicate studies, all identified articles were screened for eligibility based on title and abstract. Because of the number of studies identified in search 2 and 3, the AI tool ASreview LAB (version 1.6.3) was used during the title and abstract screening to enhance efficiency [12]. ASreview uses AI to screen and sort large amounts of publications on relevance by learning from the labeling process. A previous study found that the title and abstract screening can be stopped after 40% of the studies in the search were labeled and when ≥20 consecutive papers were labeled irrelevant; the remaining articles were then deemed as likely irrelevant [13]. To validate the ASreview selection by the reviewers, several articles known to be relevant for search 2 and 3 were verified for their inclusion by the tool. Next, the eligibility of full-text papers was examined. When a systematic review was identified, the review itself was excluded from this scoping review, but its references were screened for studies that potentially met the inclusion criteria. In that case, the study was included accordingly. The final selection was discussed among the reviewers and any disagreements were resolved.

### Data extraction and result synthesis

Data extraction forms were developed by 2 reviewers (JHBH and CBC). Variables extracted from the articles included article characteristics (e.g. journal), study characteristics (e.g. objective, study design, population size, dietary assessment method, statistical method), exposure characteristics (food biodiversity metric), and outcome characteristics (diet quality metric, health outcomes, environmental impact indicators, and corresponding statistical associations). Results were synthesized based on study outcomes. A descriptive approach was used to compare study methods and explain how food biodiversity relates to diet quality, health outcomes, and environmental impact.

## Results

### Identified papers on food biodiversity

**FIGURE 1:**
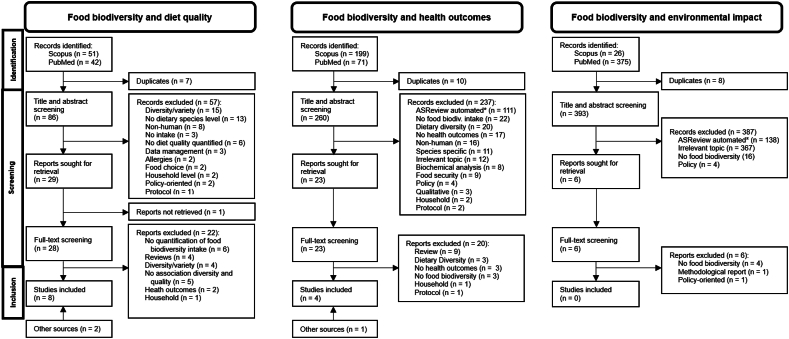
PRISMA charts for the systematic selection of articles. Left: selection of studies regarding food biodiversity and diet quality. Middle: selection of studies regarding food biodiversity and health outcomes. Right: selection of studies regarding food biodiversity and environmental impact. ∗Using AI tool ASreview. Other sources refer to articles published after 31 December, 2024 that were identified as relevant.

**FIGURE 2:**
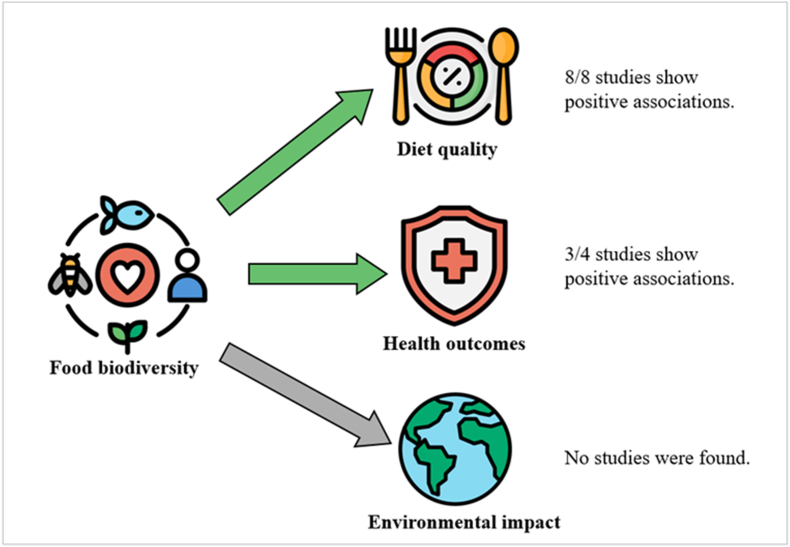
Overview of the key findings found on the associations between food biodiversity and diet quality, health outcomes, and environmental impact.

The article selection procedure for each of the 3 search strategies is presented in a PRISMA chart (Figure 1). Eight articles studying the association between food biodiversity and diet quality were identified [[14], [15], [16], [17], [18], [19], [20], [21]]. Four studies were identified studying the association between food biodiversity and health outcomes [18,20,22,23]. No studies were identified addressing the relation between food biodiversity and environmental impact. Searching the references of identified reviews did not yield any additional studies. A visual overview of the main outcomes is presented in Figure 2.

**FIGURE 3:**
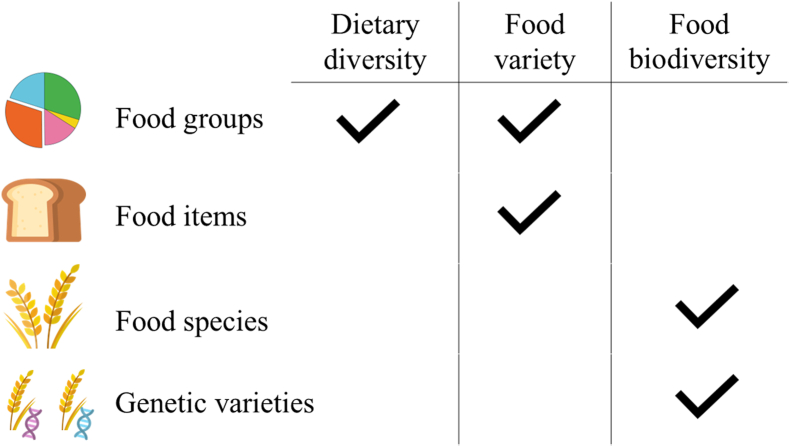
Differences in the level of specificity when assessing consumption related to dietary diversity and food biodiversity. Dietary diversity and food variety typically consider food groups, or food items, including food mixtures. Food biodiversity considers the diversity of consumed species and genetic varieties between species.

In the identified studies on diet quality and health, different metrics were used to quantify food biodiversity. A definition of each, and a description of their calculation is given in Box 1 [14,16,20,[24], [25], [26], [27], [28], [29]] for the metrics Nutritional Functional Diversity score (NFD), Dietary Species Richness (DSR), Simpson Diversity Index (SDI), Shannon Diversity Index (SHDI), Berger-Parker index (BP), and Hill numbers computed for the latter 4 food biodiversity metrics. The DSR was used in 9 studies [14,15,[17], [18], [19], [20], [21], [22], [23]], the NFD was used in 2 studies [14,16], and the SDI in 1 study [14]. One study used DSR, SDI, SHDI, and BP to calculate Hill numbers to enable unification and comparison of the different metrics [20]. In addition to food biodiversity metrics, the dietary diversity metric Food Variety Score (FVS) was also used in 1 of the studies [16]. Food biodiversity metrics and dietary diversity metrics differ regarding the level of specificity (e.g. food species compared with food groups, respectively) at which consumption is considered (Figure 3). FVS counts the number of foods (including individual foods, food mixtures, food categories or a combination of these, but not food species) consumed over a given period [30]. To use these food biodiversity metrics, different dietary intake assessment methods were applied across studies. Three studies used a single 24-h recall [14,17,20], 3 studies used a repeated 24-h recall [15,18,19], 4 studies used a Food Frequency Questionnaire (FFQ) [16,20,22,23], and 1 study used a 4-d diet diary [21].

Box 1 **Food Biodiversity Metrics Identified in this Review**

**Dietary Species Richness (DSR)**

DSR is a food biodiversity metric that counts the number of unique plant and animal species consumed during a specified period [14]. Each food ingredient should be identified at the species level. There is no minimum quantity or frequency of consumption required before a species can count toward DSR.

**Nutritional Functional Diversity (NFD)**

NFD is a food biodiversity metric that has been adapted by Di Maso et al. [16], from ecological application to measure nutritional disparity [24]. The nutritional dissimilarity of foods is calculated using a food dendrogram constructed from a set of nutrients. Nutritionally similar foods are clustered together, as opposed to nutritionally dissimilar foods. The NFD score is the ratio of the total arm length of consumed foods to the total dendrogram length, with higher scores indicating more nutritionally diverse diets.

**Simpson Diversity Index (SDI)**

SDI is a food biodiversity metric adapted from ecological studies, where it is used to quantify ecological species diversity [25]. In nutritional studies, the SDI quantifies the number of unique food species and the evenness (i.e. if species are consumed in equal proportions or not) [14]. The studies in this review used the Gini-Simpson index, with higher values indicating greater diversity [14]. In other words, the SDI measures the probability that 2 random diet species are different species [26].

**Shannon Diversity Index (SHDI)**

SHDI is a food biodiversity metric based on entropy, measuring the number of unique species and their relative quantities [27]. This metric is more biased toward evenness than richness by giving more significance to species consumed in larger quantities.

**Berger-Parker Index (BP)**

BP is a food biodiversity metric based on the proportion of the dominant, or most abundant, species in a diet [28]. Values close to 1 represent high dominance by this one species and thus less evenness, whereas values close to 0 indicate greater proportional evenness between the species. When taking the reciprocal, higher values represent more diversity.

**Hill numbers**

Also known as effective species indices, it could help unify food biodiversity metrics by using the same functional unit [20,29]. Hill numbers represent the number of distinct species consumed if the diet were perfectly even or when species are consumed in equal relative quantities. Proportions of species intake can be assessed based on weight (g/d) or energy (kcal/d).

Alt-text: Box 1

### Food biodiversity and diet quality

The structured search identified 8 studies that investigated the association between food biodiversity and diet quality (Table 2). Each of these studies used a different approach to assess food biodiversity, diet quality, or their associations. Diet quality metrics used in these studies were based on micronutrient adequacy [Mean Adequacy Ratio (MAR), Mean Probability of Adequacy, and the Nutrient-Rich Foods score (NRF-8.3)], or on adherence to dietary guidelines [Dutch Healthy Diet Index 2015 (DHD15), Mediterranean Diet Score (MDS), and Healthy Eating Index 2015 (HEI-2015)]. All studies showed a small but significant positive association between the food biodiversity and diet quality metrics used (Table 2). Three studies that included multiple food (bio)diversity metrics in their analysis [14,16,20] and 2 studies evaluating the dietary assessment period and quantity of intake [15,21] will be shortly described.

**TABLE 2:** Summary of articles relating food biodiversity to diet quality outcomes.

Authors	Population, region	Study design	Dietary assessment method	Food biodiversity metric	Diet quality metric	Type of analysis	Adjustment	Association food biodiversity and diet quality	Species intake (period)
Aceves-Martins et al. [21]	3558 adults from NDNS 9–11 (2016–2019), United Kingdom	Cross-sectional	Four-d food intake diary	DSR	NRF-8.3 (scores ranged between 0 and 5000)	Fitting regression analysis	Age, household income, and Index of multiple deprivation (IMD)	Positive association of DSR with NRF 8.3: Estimate: 159.3, SE: 8.71, *t =* 18.13, *P* < 0.001	Total 269 species identified, median DSR 49 (Q1 = 43, Q3 = 56, range 14–92)
Bakker et al. [15]	2078 adults (19–79 y), The Netherlands	Cross-sectional	24-h recall (2 nonconsecutive days)	DSR	DHD15 index (score 0–140)	Linear regression	Sex, education, and mean energy intake. Additionally, (quantity of fruit, and vegetable intake in g/d)	Positive association of DSR to DHD15 index: β: 1.40, 95% CI: [1.25, 1.55], *P* < 0.001 DSR fruit: β: 4.01 [3.65, 4.38] DSR vegetable: β: 0.84 [0.61, 1.08] Additionally adjusted for quantity: DSR fruit: β 1.91 [1.40, 2.42] *P* < 0.001 DSR vegetable: β: 0.16 [−0.07, 0.40]	Total 157 species identified, mean 13 (SD: 4.55) (2 d)
Di Maso et al. [16]	7948 adults (no age range given), controls from a cancer case-control study, Italy	Cross-sectional	70 item FFQ (represent 1-y habitual intake)	NFD (28 nutrients), FVS (food item count in a week)	MDS (score 0–9) (grouped as 0–2, 3–6, and 7–9 points), HEI (scale of 0–100) (grouped as <63.5, 63.5–<69.1, and >69.1 points)	Multinomial logistic regression models	Sex, age, region, education, year of enrolment, BMI, physical activity, smoking, and energy intake	Adjusted OR [95% CI] of positive association of MDS 7–9 points with Q4 NFD: 11.26, 95% CI: [7.88, 16.09] MDS (7–9 points) with Q4 FVS: 6.80 [4.84, 9.54]. HEI-2015 (≥69.1) with Q4 NFD: 2.86 [2.39, 3.42]; HEI-2015 (≥69.1) with Q4 FVS: 2.72 [2.27, 3.26]	NA
Hanley-Cook et al. [20]	2148 women (18–90 y) from Democratic republic of Congo, Ecuador, Kenya, Sri Lanka and Viet Nam, referred to as the LMIC study	Cross-sectional	Multiple pass 24-h recall	Hill numbers for DSR, SHDI, SDI, and BP	MAR (in percentage points, based on 6 micronutrients)	Linear mixed-effects model	Not described	Positive associations between Hill numbers based on weight-intake and MAR in percentage points (Q4 vs. Q1) (β [95% CI]), *P* < 0.001 for all: Hill-DSR: 12.1 [10.4, 13.8] Hill-SHDI: 13.7 [12.2, 15.3] Hill-SDI: 12.6 [11.1, 14.1] Hill-BP: 11.0 [9.53, 12.6]	Not described
Lachat et al. [14]	6226 participants (34% women, mean age 31.0 ± 11.7; children 6–24 mo old) from rural Benin, Cameroon, Democratic Republic of Congo, Ecuador, Kenya, Sri Lanka, and Vietnam	Cross-sectional	24-h dietary recall	DSR, Gini-Simpsons index of diversity (SDI), NFD	MAR (score 0–1, based on 6 micronutrients)	Mixed-effects linear regression model. Fixed: season Random: country	Energy intake	Significant (all β coefficients are *P <* 0.001) positive unstandardized association between MAR and DSR (β: 0.03, 0.001 SE) MAR and SDI (β: 0.70, 0.02 SE) MAR and NFD (β: 0.52, 0.02 SE) Standardized associations MAR and DSR (β: 0.10, 0.003 SE) MAR and SDI (β: 0.08, 0.08 SE) MAR and NFD (β: 0.06, 0.002 SE)	Total 234 species identified, mean 10.19 (SD: 3.52) for women (1 d)
Loukrakpam et al. [17]	1961 women (15–49 y), India	Cross-sectional	24-h recall	DSR	MPA (score 0–1, based on 12 micronutrients)	Logistic regression, chi-squared test	Age and sex	Positive association between DSR ≤13 with MPA < 0.5 Adjusted OR 1.88, CI: 95% [1.3, 2.72] *P* < 0.001	Mean 13.55 (SD: 2.17) species for woman (1 d)
Penafiel et al. [19]	178 Rural, indigenous adult women (18–69 y), Ecuador	Cross-sectional	24-h recall, repeated after 14 d	DSR	MAR (score 0–1, based on 9 nutrients)	Linear quantile mixed model	Age, occupation, and education as fixed effects; village as a random effect	Positive association of DSR with MAR Estimate: 0.019, *P <* 0.001	Total 119 species identified, median 16 (inter quartile range: 4. Range 8–24) (2 d)
Vogliano et al. [18]	30 women (18–50 y), Solomon Islands	Cross-sectional	24-h recall (2 nonconsecutive days)	DSR	MAR (score 0–1, based on 10 nutrients)	Linear regression	Energy intake	Positive correlation between DSR with MAR β: 0.039,*P* < 0.05	Total 87 species identified, mean 12.1 (SD: 3.4) (2 d)

Abbreviations: BP, Berger-Parker index; CI, confidence interval; DHD15, Dutch Healthy Diet 2015; DSR, Dietary Species Richness; FFQ, food frequency questionnaire; FVS, Food Variety Score; HEI-2015, Healthy Eating Index 2015; LMIC, low-middle income country; MAR, Mean Adequacy Ratio; MDS, Mediterranean Diet Score; MPA, Mean Probability of Adequacy; NFD, Nutritional Functional Diversity; NRF-8.3, Nutrient-Rich Food index 8.3; OR, odds ratio; SDI, Simpson Diversity Index; SHDI, Shannon Diversity Index.

The study by Lachat et al. [14] compared how the food biodiversity metrics DSR, NFD, and SDI relate to the MAR, which serves as a proxy for micronutrient adequacy. They used dietary intake data from women and children in 7 LMICs. The authors standardized the associations of the different food biodiversity metrics with the MAR to allow for comparison. DSR showed the strongest association [β: 0.10, (0.003 SE)] with MAR, compared with SDI [β: 0.08, (0.08 SE)] and NFD [β: 0.06, (0.002 SE)] (Table 2). On the basis of these outcomes, Lachat et al. [14] recommended DSR as the most appropriate metric to measure food biodiversity.

The study carried out by Di Maso et al. [16] among Italian adults found that individuals with higher MDSs showed stronger associations with higher NFD scores [odds ratio (OR): 11.26, 95% confidence interval (CI): (7.88, 16.09)], than with higher FVS [OR: 6.80, (4.84, 9.54)] (Table 2). Similarly, the HEI-2015 was more strongly associated with higher NFD scores than with higher FVS (Table 2). Subsequently, they suggested that NFD is better at capturing the dimension of diversity as part of diet quality, as it reflects both the number and the nutritional dissimilarity of the foods consumed.

A recent study evaluated how 4 food biodiversity metrics (DSR, SHDI, SDI, and BP) relate to MAR outcomes, using cross-sectional data from women across 5 LMICs [20]. As explained in Box 1, Hill numbers were calculated for these metrics based on weight or energy intake to unify them and allow comparability. Both weight- and energy-based Hill numbers were positively associated with MAR outcomes for all food biodiversity metrics, but the strongest association was found for weight-based Hill-SHDI [Q4 compared with Q1 β: 13.7, 95% CI: (12.2, 15.3)] (Table 2). In addition, they compared the ability of these metrics to differentiate the number of effective species between 14 countries. Use of the Hill-DSR number resulted in the best discrimination between countries and between individuals within the same country, compared with the other Hill numbers.

Another recent study evaluated whether a 4-d diary can capture DSR and its association to diet quality among adults in the United Kingdom (Table 2) [21]. They found that the first 2 d of a 4-d diary captured 80% of the number of dietary species recorded. Moreover, they found a small but significant positive association between DSR and the NRF-8.3 score, which serves as a proxy for diet quality.

Similarly, a study among Dutch adults found that higher DSR scores were significantly associated with slight increases in diet quality, as measured by the DHD15 [β: 1.40, 95% CI: (1.25, 1.55)] (Table 2) [15]. They did not quantify overall DSR, because certain foods were excluded (e.g. condiments, spices, sauces, and other highly processed foods). The associations for DSR fruit and DSR vegetables with DHD15 [DSR fruit: β: 4.01 (3.65, 4.38), DSR vegetable: β: 0.84 (0.61, 1.08)] became attenuated after adjusting for quantity (g/d) [DSR fruit: β: 1.91 (1.40, 2.42), DSR vegetable: β: 0.16 (−0.07, 0.40)].

### Food biodiversity and health outcomes

Four studies assessed the association between food biodiversity and health outcomes. Three studies used data from the European prospective cohort study European Perspective Investigation into Cancer and Nutrition (EPIC) to investigate the association between species intake per year (DSR) and mortality [20,22], and gastrointestinal cancer [23]. The third study described the cross-sectional association between DSR and body fat percentage [18].

DSR was independently inversely associated with total mortality and mortality because of cancer, cardiovascular disease, respiratory disease, and digestive disease (Table 3) [22]. A 10-species increment in DSR per year showed an inverse association with total mortality in the multiadjusted model [hazard ratio (HR): 0.90 (95% CI: 0.89, 0.90)]. Furthermore, a 10-species increment in DSR showed an inverse association [HR (95% CI)] with cause-specific mortality from digestive disease [0.80 (0.76, 0.86)], respiratory disease [0.84 (0.80, 0.88)], cardiovascular disease [0.88 (0.86, 0.90)], and cancer [0.93 (0.92, 0.95)]. After excluding the lowest 5% and 10% of species intake (g/d) from each EPIC food (sub)group, the findings remained similar regarding direction, strength, and trend of association. In addition, they found that individuals in the highest DSR quintile consumed relatively more species from the food groups vegetables, fruit, nuts and seeds, and condiments, compared with the lowest quintile.

**TABLE 3:** Summary of articles relating food biodiversity to health outcomes.

Authors	Population, region	Study design	Dietary assessment method	Food biodiversity metric	Health outcomes	Type of analysis	Adjustment	Associations of food biodiversity with health outcomes	Species intake (period)
Hanley-Cook et al. [20]	451,390 adults (25–70 y) from 9 European countries, from the European Perspective Investigation into Cancer and Nutrition (EPIC) cohort study	Longitudinal	Included self- or interviewer-administered semiquantitative FFQs. EPIC food composition database comprises >11,000 food and beverage items. Country-specific dietary questionnaires	Hill numbers for DSR, SHDI, SDI, and BG	All-cause mortality	Multivariable-adjusted Cox proportional hazard regression models	Multiadjusted model: smoking status, educational level, marital status, physical activity, alcohol intake, total energy intake, Mediterranean diet score, red, and processed meat intake, fiber intake	Inverse associations between Hill numbers based on weight-intake and all-cause mortality (Q5 vs. Q1) (HR [95% CI]), *P* < 0.001 for all: Hill-DSR: 0.66 [0.62, 0.69] Hill-SHDI: 0.82 [0.79, 0.85] Hill-SDI: 0.84 [0.81, 0.88] Hill-BP: 0.86 [0.83, 0.89]	Not described
Hanley-Cook et al. [22]	451,390 adults (25–70 y) from 9 European countries, from the European Perspective Investigation into Cancer and Nutrition (EPIC) cohort study	Longitudinal	Included self- or interviewer-administered semiquantitative FFQs. EPIC food composition database comprises >11,000 food and beverage items. Country-specific dietary questionnaires	DSR	Total mortality, cause-specific mortality (cancer, heart disease, respiratory disease, digestive disease)	Multivariable-adjusted Cox proportional hazards regression models. With age as the primary underlying time variable	Multiadjusted model: smoking status, study center, educational level, marital status, physical activity, alcohol intake, total energy intake, Mediterranean diet score, red, and processed meat intake, fiber intake	Inverse association between 10-species DSR increment per year with total mortality HR: 0.90 [95% CI: 0.89, 0.90] *P* < 0.001]. Inverse association between 10-species increment and cause-specific morality (HR [95% CI]) all *P* < 0.001: digestive disease (0.80 [0.76, 0.86]), respiratory disease (0.84 [0.80, 0.88]), heart disease (0.88 [0.86, 0.90]), and cancer (0.93 [0.92, 0.95]); 10-species DSR increment and association with mortality rates per gender: Men (HR: 0.84 [0.83, 0.86]), and women (HR: 0.93 [95% CI: 0.92, 0.94])	Median 68 species (1 y); Q1: <48, Q2: 48–64, Q3: 64–72, Q4: 72–81, Q5: >81
Huybrechts et al. [23]	451,390 adults (25–70 y) from 9 European countries, from the European Perspective Investigation into Cancer and Nutrition (EPIC) cohort study	Longitudinal	Included self- or interviewer-administered semiquantitative FFQs. EPIC food composition database comprises >11,000 food and beverage items. Country-specific dietary questionnaires	DSR	Gastrointestinal cancer (overall and site-specific; esophageal, proximal colon, colorectal, liver)	Multivariable Cox proportional hazards regression models	Multiadjusted model: age, sex, study center, smoking status, educational level, marital status, physical activity, alcohol intake, BMI, total energy intake, calcium intake, Mediterranean diet score, red, and processed meat consumption, fiber intake	Inverse association between 10-species DSR increment per year and overall GI cancer risk HR: 0.94 [95% CI: 0.92, 0.97], *P* < 0.0001. Per 10-species increment, the HR for site-specific cancer, *P* < 0.015 for all: Esophageal squamous cell carcinoma: 0.98 [0.96, 0.99], colorectal cancer: 0.96 [0.93, 0.99], liver cancer: 0.79 [0.71, 0.89] Association between 10-species increment in DSR animal/plant and overall gastrointestinal cancer risk, both *P* <0.002): DSR animal_:_ HR: 0.98 (95% CI: 0.96, 0.99); DSR plant: HR: 0.93 (95% CI: 0.90, 0.97) No linear trend DSR and energy intake	Median 68 species (1 y); Q1: <48, Q2: 48–64, Q3: 64–72, Q4: 72–81, Q5: >81
Vogliano et al. [18]	30 women (18–50 y), Solomon Islands	Cross-sectional	Multiple pass 24-h recall (2 nonconsecutive days), focus group discussions	DSR, FVS (food items). DDS	Diet quality, anthropometric measures (height, weight, fat percentage)	Linear regression analysis	Energy intake, household income, food security status	Adjusted inverse associations between DSR, FVS, DDS, and body fat percentage: DSR: *R*^2^: 0.223, β: –1.36,*P* = 0.068; DDS: *R*^2^: 0.248, β: –3.21,*P* = 0.040; FVS: *R*^2^: 0.262, β: –1.44, *P* = 0.030	Identified 221 edible species, of which 87 were consumed, mean DSR 12.1 (SD: 3.4) (1 d)

Abbreviations: BP, Berger-Parker index; CI, confidence interval; DDS, Dietary Diversity Score; DSR, Dietary Species Richness; FFQ, food frequency questionnaire; FVS, Food Variety Score; HR, hazard ratio; SDI, Simpson Diversity Index; SHDI, Shannon Diversity Index.

When comparing the associations of 4 food biodiversity metrics (DSR, SHDI, SDI, and BP) by calculating Hill numbers with all-cause mortality, higher quintiles of each of the Hill numbers were associated with lower all-cause mortality rates over a 20-y follow-up [20]. Hill-DSR showed the strongest association with a reduction in mortality rates when comparing Q1 with Q5 [HR: 0.66 (95% CI: 0.62, 0.69)], but the highest absolute reduction in mortality rates was found for Hill-SHDI. However, these associations were not adjusted for confounders. When dietary risk factors were excluded from the models, assuming them to be mediators, the associations became stronger. Results were similar between weight-based and energy-based Hill numbers.

Huybrechts et al. [23] showed that a 10-species increase per year in DSR had a small but significant inverse association with overall gastrointestinal cancer risk [HR: 0.94 (95% CI: 0.92, 0.96)] (Table 3). Similarly, statistically significant inverse associations were found between a 10-species increase per year in DSR and some specific gastrointestinal cancer types, including esophageal squamous cell carcinoma, colorectal cancer, and liver cancer (Table 3). Furthermore, BMI appeared to modify the effect of DSR on gastrointestinal cancer risk. When the association was stratified for BMI, the normal and overweight groups showed similar significant inverse associations across the quintiles, whereas this association was nonsignificant for individuals who were obese (BMI >30 kg/m^2^). When considering 10-species increments in DSR from plant-based or animal-based foods separately, small but significant inverse associations with overall gastrointestinal cancer risk were found [DSR plant—HR: 0.93 (95% CI: 0.90, 0.97); DSR animal—HR: 0.98 (95% CI: 0.96, 0.99)] (Table 3). However, the associations could not be explained by DSR within a single food group (e.g. DSR fruit). Therefore, the authors suggest that the protective effect of DSR can be explained by a cumulative effect of overall DSR [22,23].

A study including 30 women of reproductive age from the Solomon Islands showed an inverse association between DSR and body fat percentage, which became statistically nonsignificant after adjusting for energy intake (Table 3) [18]. This indicates that there is weak evidence that food biodiversity is inversely associated with body fat percentage.

## Discussion

To our knowledge, this is the first systematic scoping review presenting an overview of available data on the association between food biodiversity and diet quality, selected health outcomes, and environmental impact. This review revealed 8 studies describing a positive association between food biodiversity and diet quality, and 4 studies describing a positive association between food biodiversity and improved health outcomes. No studies were identified reporting on the association between food biodiversity and environmental impact. The results of the identified studies could not be compared quantitatively because of differences in dietary assessment methods, food biodiversity and diet quality metrics, and covariate adjustment. Nevertheless, all available studies showed significant positive associations between food biodiversity metrics and diet quality and a reduced risk of mortality or gastrointestinal cancers. The results underline the potential improvement of human health through increasing food biodiversity intake, possibly by improving overall diet quality.

### Is food biodiversity linked to diet quality and human and environmental health?

All studies identified in this review showed that higher food biodiversity intake was associated with better diet quality and health outcomes, but the effect sizes were relatively small. For example, for every additional unique species consumed over 2 d, the DHD15 index increased only 1.40 points (on a scale of 0–140) for Dutch adults [15]. Similarly, a higher DSR was associated with a higher NRF-8.3 score as a proxy for diet quality, but the effect estimate was small [21]. Studies found that DSR for food subgroups, like fruit or vegetables, did not show significant associations with diet quality [15], mortality [22], and gastrointestinal cancer risk [23]. This may be due to insufficient statistical power to detect effect sizes even smaller than those observed for total DSR. Pooled data from multiple studies may be more suitable for subgroup analysis. Therefore, it is crucial that future studies use consistent methodologies, including nutritional assessment methods and diversity scores, to allow for estimate pooling.

Very recent studies add to the evidence of the positive association between food biodiversity intake and diet quality and health outcomes. One study shows a moderate correlation between food biodiversity (DSR) and diet quality (adherence to the Mediterranean diet), as well as a protective association with mortality in older Spanish adults [31]. A second study in Italian adults showed that total DSR, and subgroups DSR fruit and DSR vegetable are associated with higher diet quality (measured with the Global Diet Quality Score) [32]. Although these studies are not included in our results because they were published after our initial submission, their findings are consistent with the studies described in this review and underscore the potential of food biodiversity intake to shape human health.

The associations between food biodiversity and diet quality and health outcomes appear to be affected by the quantity of intake, especially by species from plant-based food groups like fruits and vegetables. Recently, Hanley-Cook et al. [20] showed that Hill-DSR was most strongly associated with lower all-cause mortality rates compared with the evenness-based Hill numbers (Hill-SHDI, Hill-SDI, and Hill-BP), which consider the proportion of species intake based on weight (g/d) or energy (kcal/d). This suggests that species consumed in low quantities (g/d) have an important additional contribution toward health and therefore should not be weighted by the proportion of intake. In contrast, another study by Hanley-Cook et al. [22] showed that excluding species consumed in the lowest quantities (5% and 10%, g/d, e.g. species and herbs) from the analysis did not change the association between DSR and mortality, suggesting that these associations are mostly driven by species consumed in higher quantities. Similarly, Bakker et al. [15] found that the associations of DSR fruit and DSR vegetables with DHD15 were attenuated after adjusting for the quantity of fruit or vegetable intake (g/d). Thus, the role of quantity (in weight) of species intake on diet quality and health is unclear, and whether this attenuation is related to the quantity of intake acting as a confounding variable or by multicollinearity between quantity and species intake. For example, the quantity of intake and DSR were highly correlated for fruit (cc >0.7), but not for vegetables (cc >0.4) [15]. A different study highlights the importance of unique species intake from “healthier” food groups, such as fruit and vegetables, as already set out in the dietary guidelines. Adherence to dietary guidelines was associated with significantly higher DSR over 4 d using UK National Diet and Nutrition Survey (NDNS) data, specifically, 7 additional species when adhering to the guideline for fiber, and 6 for fruits and vegetables [21]. Furthermore, an increased consumption of plant-based species, compared with animal species, showed a stronger association with reduced gastrointestinal cancer risk, highlighting the potential benefits of “healthier” food groups [23]. Nevertheless, no single DSR subgroup (e.g. DSR fruit) was found to be responsible for this association, suggesting that the protective effect of DSR is to be explained by a cumulative effect. Taken together, these findings show that it is still unclear whether the associations between food biodiversity, diet quality, and health outcomes are explained by adherence to dietary guidelines, such as quantity of intake and plant-based foods consumption, or whether these findings are affected by the suggested multicollinearity. Therefore, future studies should be well designed with sufficient statistical power to detect potential small effect sizes. In addition, the role of quantity (in weight) of the species consumed and their respective food group categorization should be evaluated, to be able to better understand the role of food species intake in relation to diet quality and health outcomes.

Clarifying the underlying mechanisms of food biodiversity contributing to improved health outcomes would help to understand the role of plant-based species and the quantity of intake. Currently, there are 4 proposed mechanisms [22]. First, the sampling effect refers to the increased probability of consuming highly nutritious foods and covering the intake of all required micronutrients when increasing the food biodiversity intake. Second, the complementary effect postulates greater beneficial effects through synergistic interactions between species. Third, minimizing trade-offs refers to the reduction of adverse effects of contaminants, residues, and pesticides, because of an overconsumption of one species. Finally, diet-induced variations in human microbial communities are suggested to contribute to resilient gut-microbiome communities and improved metabolic health [33]. However, because none of the suggested mechanisms has been confirmed on a species level, future studies should focus on elucidating the underlying mechanisms linking food biodiversity to health outcomes.

In our search, no studies were identified that explored the association between food biodiversity and the environmental impact of food consumption. A very recent of study of Berden et al. [34] that was published after our search date is, to our knowledge, the first to investigate the relation between food biodiversity and greenhouse gas emission and land use. They found inverse associations between DSR_Plant_ and greenhouse gas emissions and land use. It is a first insight in our key understanding of the ecological impacts of increasing food biodiversity to avoid unintended consequences, and to understand if food biodiversity can play a role in food systems that are respectful of the planetary boundaries. The proportion of plant- and animal-based species consumption is important because animal-based products, such as beef, generally have a larger environmental impact [35]. One could speculate that when promoting food biodiversity by increasing the intake of plant-based species, while simultaneously reducing animal-based species intake, the overall dietary environmental impact would be lower, provided that energy intake remains constant. However, depending on the origin of the consumed plant-species and how they are produced, the environmental impact of their consumption can vary. For instance, consuming products that are locally produced with alternative agricultural practices can be beneficial (e.g. minimum tillage, cover crops), as most of these practices are more biodiverse-friendly than intensive practices [36]. Moreover, as already pointed out by Hanley-Cook et al. [10], the relationship between biodiversity in farms, nature, and food biodiversity is not straightforward and does not guarantee diet quality. To study these complex relationships, it is essential to use or develop a suitable metric. Future studies need to identify how food biodiversity intake is related to various environmental aspects, including greenhouse gas emissions, land use, water use, biodiversity loss, as well as potential trade-offs. Additionally, it should be explored if food biodiversity can contribute to food systems respectful of the planetary boundaries, using a suitable metric to relate these factors.

### Measuring food biodiversity

Among the various food biodiversity metrics in the reviewed studies, DSR is the most widely used metric. However, it is currently unclear if DSR is indeed the most relevant. There are multiple metrics available to quantify biodiversity in addition to the ones included in this scoping review, which have previously been reviewed [26]. The included studies used 1 or multiple of the following metrics to quantify food biodiversity: NFD, DSR, SDI, SHDI, BP, or calculated Hill numbers for the latter 4. Two studies showed that DSR was more strongly related to micronutrient adequacy, compared with evenness-based metrics (SDI, SHDI, and BP) [14,20]. Evenness may be desirable in an ecological setting, but less so from a nutritional perspective, where healthy foods should be consumed in higher quantities than unhealthy foods. However, calculating ideal evenness proportions or using diverging weighing factors of species within food groups could hamper comparability between studies. For application in nutrition sciences, adaptations to the SDI have been made to favor healthy food consumption over equal consumption, but these adaptations shifted the focus from species to food groups, making it unsuitable for quantifying food biodiversity [37,38]. Given that evenness is a key aspect of ecological biodiversity, the role of evenness in a food biodiversity metric should not be disregarded, as it might be used to study connections between human, ecological, and planetary health. Evaluating the suitability of different food biodiversity metrics within a single study requires standardizing the outcomes and explicitly documenting the standardization procedure. Hanley-Cook et al. [20] compared 4 food biodiversity metrics by calculating Hill numbers and found that the 3 evenness-based metrics (Hill-SHDI, Hill-SDI, and Hill-BP) were moderately to strongly correlated (Spearman’s rank ρ: 0.79–0.97). Hill-DSR was only weakly correlated with the other Hill numbers (ρ: 0.04–0.22). In addition, they found that Hill-DSR is more sensitive to changes in species intake when comparing countries with different dietary patterns, compared with the evenness-based Hill numbers [20]. Although this unification is an important step toward comparability, the interpretation of relative abundances represented by Hill numbers remains challenging from a nutritional perspective. It should be noted that Hanley-Cook et al. [20] did not include metrics based on functional diversity in this comparison. NFD allows insight into the level of similarity between species, but is more complex to calculate because it requires nutritional composition data for each species. Lachat et al. [14] did include NFD, but still argued for the use of the DSR metric over NFD and SDI, because of its straightforward calculation and stronger association with micronutrient adequacy. In short, DSR is the easiest metric to calculate and interpret, with strong validity through its associations with micronutrient adequacy, reduced mortality, and better discriminatory characteristics compared with the evenness-sensitive Hill numbers. Therefore, we concur with previous studies that DSR is the most feasible metric to evaluate food biodiversity across contexts and time. However, Di Maso et al. [16] preferred the use of NFD because of its ability to include nutrient content dissimilarity between species and its positive association with the MDS. Because DSR and NFD metrics measure fundamentally different aspects of diversity (see Box 1), they cannot replace each other. Alternatively, the combined use of complementary metrics (e.g. DSR and NFD) could provide insights into multiple dimensions of food biodiversity (e.g. richness and disparity). In line with others, we advocate for the proper and deliberate use of different quantitative food biodiversity metrics based on the aim of the research [26,39]. Distinct use of the various food biodiversity metrics should be considered, as opposed to 1 all-encompassing metric, which could obscure the underlying differences when multiple dimensions of diversity are combined into 1 output.

The type of dietary assessment method used to quantify food biodiversity determines the quality of dietary information, and thus the accuracy of the food biodiversity metric. The identified articles in this review used either a single or repeated 24-h recall, diary, or FFQ. When mapping the number and type of species consumed, an open-ended assessment method is considered most appropriate (e.g. diary or 24-h recall), as it does not require predefining and limiting the maximum number of species to include (e.g. FFQ). FFQs often aggregate food items based on nutrient content, hampering species identification and thus potentially allowing misclassification. The number of identified food species is inherently dependent on the period over which consumption is measured. One study showed that the first 2 d of a 4-d open-ended assessment period covered 80% of the unique species consumed in a Western-style diet [21], but including more days better accounts for within-person variation in food species intake. Therefore, a minimum of 2 (nonconsecutive) 24-h food diaries or recalls may be considered appropriate to quantify the number of species for a Western-style diet. However, for dietary patterns from other regions, it is still unclear how many dairy or recall days are needed to capture the majority of habitual species intake.

### Limitations

This review has some limitations. During the study selection process, studies were not assessed for quality, as this scoping review aimed to provide a descriptive overview. The transparency and reproducibility of this review are ensured by adhering to the PRISMA-ScR method, which does not require a critical appraisal step [11]. The use of a critical appraisal step in scoping reviews is still debated [40]. In this review, only peer-reviewed articles were included to ensure a minimum quality standard. The majority of the included studies used cross-sectional designs, which inherently limit interpreting the temporality and causality of food biodiversity intake concerning changes in diet quality and the onset of health outcomes. Although a set of longitudinal studies provides insight into temporal associations, such as disease onset and mortality, their observational nature limits drawing conclusions about causality and should therefore be interpreted with caution. Randomized control trials are needed to support these observations. In addition, included studies vary in population size (30–7948), and differences in age and nationality limit the generalizability of findings to the broader global population. Furthermore, the use of AI tool ASreview during the search on associations between food biodiversity and health outcomes may introduce a risk of missing relevant articles. Nonetheless, the impact is expected to be only minimal, as the inclusion of certain preselected studies was verified by the authors, and reference searching was used to identify additional relevant articles.

## Conclusion and future recommendations

The current scoping review indicates that food biodiversity is positively associated with diet quality and beneficial health outcomes, and points out several considerations for future research. Eight studies on the association between food biodiversity and diet quality were identified, and 4 on food biodiversity and health outcomes. The absence of studies assessing the association between food biodiversity and environmental impact outcomes indicates that research is especially needed to explore these links. Future studies should be well designed with sufficient statistical power to provide good-quality evidence to investigate the underlying mechanism of the health benefits associated with increased food biodiversity intake, as well as potential trade-offs. Currently, DSR can be considered the most feasible metric to quantify food biodiversity. To cover other dimensions of food biodiversity, DSR can be used in combination with other metrics. In addition, food biodiversity metrics should be further validated, or new ones developed that allow us to study the relations between food biodiversity intake and its health effects with environmental impact. To accurately assess food species consumption, a 24-h recall or diary of ≥2 (nonconsecutive) days seems most suitable. In addition, it is of interest to understand the linearity of the relationship between food biodiversity and diet quality, human health, and planetary health. The majority of existing dietary guidelines consider dietary diversity between and within food groups to ensure diet quality and health for a population [41]. Dietary diversity metrics, such as the Minimal Dietary-Diversity Index, are widely used as a proxy for micronutrient adequacy in low-income countries. To complement such metrics, future initiatives should quantify diversity between and within food groups as well as on a species level within one study, and relate these to diet quality and health outcomes in high-, middle-, and low-income countries. This should help to explore the added value of food biodiversity over dietary diversity for human and planetary health, as this is currently not clear.

## Author contributions

The authors’ responsibilities were as follows – JHBH, MD-K, AW: designed the research; JHBH, CBC: conducted the research; JHBH, CBC: analyzed the data; JHBH, CBC: wrote the paper; MD-K, AW, CvD, EJMF, BdR, SB: critically reflected on the content; JHBH: had primary responsibility for the final content; and all authors: have read and approved the manuscript.

## Data availability

Data described in the manuscript can be made available upon reasonable request.

## Declaration of Generative AI and AI-assisted technologies in the writing process

During the preparation of this work, the author(s) used Co-Pilot to improve readability and language. After using this tool/service, the author(s) reviewed and edited the content as needed and take(s) full responsibility for the content of the publication.

## Funding

No funding was received for this study.

## Conflict of interest

BdR reports a relationship with University of Aberdeen that includes board membership, funding grants, and travel reimbursement. CvD reports a relationship with World Wide Fund For Nature that includes employment. All other authors report no conflicts of interest..
