# Supplementary material for: Food Biodiversity and its Association with Diet Quality and Health Outcomes-A Scoping Review
Source: Adv Nutr. 2025 Nov 1;16(12):100551. doi: 10.1016/j.advnut.2025.100551 (PMC12677162; doi:10.1016/j.advnut.2025.100551)
Supplement: Multimedia component 1 [file mmc1.docx]

**Food Biodiversity and its Association with Diet Quality and Health Outcomes - a Scoping Review**

Jinke Baan Hofman & Celia Bannenberg Cavero

**Supplementary data**Intended for publication

Supplementary Table 1. Search strategies by objective and database (articles published prior to and including 31 December 2024).

| Addressing question | Database | Search strategy |
| --- | --- | --- |
| 1) Food biodiversity and diet quality | Scopus | TITLE-ABS-KEY ( ( "food biodivers*" OR "diet* biodivers*" OR "nutri* biodivers" OR agrobiodiversity OR "dietary species richness" OR "nutri* functional divers*" OR "ecological biodivers*" ) AND ( "diet quality" OR "nutri* quality" OR "healthy eating index" OR "diet* intake" OR "nutri* intake" OR "nutri* adequacy" OR "nutri* density" ) ) AND ( LIMIT-TO ( DOCTYPE , "ar" ) ) |
|  | PubMed central | ("food biodivers*" OR "diet* biodivers*" OR "nutri* biodivers" OR "dietary species richness" OR "nutri* functional divers*") AND ("diet quality" OR "nutri* quality" OR "healthy eating index" OR "diet* intake" OR "nutri* intake" OR "nutri* adequacy" OR "nutri* density") |
| 2) Food biodiversity and health outcomes † | Scopus | TITLE-ABS-KEY ( ( "food biodivers*" OR "diet* biodivers*" OR "nutri* biodivers" OR agrobiodiversity OR "dietary species richness" OR "nutri* functional *divers*" ) AND ( "health outcome*" OR disease OR mortality OR morbidity OR "hypertension" OR anemia OR "disability" OR "public health" OR "health impact" OR "incidence" OR "risk factor" OR obesity OR cancer OR inflamma* OR immun* OR cognit* OR stunting OR kidney OR "gut microb*")) |
|  | PubMed central | ("food biodivers*" OR "diet* biodivers*" OR "nutri* biodivers*" OR "dietary species richness" OR "nutri* functional *divers*") AND ("health outcome*" OR disease OR mortality OR morbidity OR "hypertension" OR anemia OR "disability" OR "public health" OR "health impact" OR "incidence" OR "risk factor" OR obesity OR cancer OR inflamma* OR immun* OR cognit* OR stunting OR kidney OR "gut microb*") |
| 3). Food biodiversity and environmental impact | Scopus | TITLE-ABS-KEY ( ( ( "food biodivers*" ) OR ( "diet* biodivers*" ) OR ( "dietary species richness" ) OR ( "nutritional functional diversity" ) ) AND ( ( "environment* impact*" ) OR ( "environment* indicator*" ) OR ( "biodiversity impact*" ) OR ( "biodiversity loss*" ) OR ( "greenhouse gas emission*" ) OR ( land use ) OR ( blue water use ) OR ( fresh water use ) OR ( eutrophication ) OR ( acidification ) ) ) |
|  | PubMed | ("food biodiversity") OR ("diet* biodiversity") OR ("dietary species richness") OR ("nutritional functional diversity")) AND (("environment* impact*") OR ("environment* indicator*") OR ("biodiversity impact*") OR ("biodiversity loss") OR ("greenhouse gas emission*") OR ("land use") OR ("blue water use") OR ("fresh water use") OR ("eutrophication") OR ("acidification")) |

† No additional articles when including any or combinations of the following terms:
anthropometric OR "disease risk" OR "non-communicable disease" OR "chronic disease" OR "cardiovascular disease" OR stroke OR cardiometabolic OR "blood pressure" OR "adjusted life years" OR "health burden" OR "life expectancy" OR diabetes OR overweight OR adiposity OR dyslipidaemia OR "blood glucose" OR respiratory OR "digestive system" OR "mental wellbeing" OR anaemia OR "linear growth" OR cholesterol OR triglyceride OR insulin OR "body weight" OR bodyweight OR renal OR nephro* OR gastrointestinal OR “gut health.
